# Hepatoprotective Effects of Kaempferol-3-*O*-α-l-Arabinopyranosyl-7-*O*-α-l-Rhamnopyranoside on d-Galactosamine and Lipopolysaccharide Caused Hepatic Failure in Mice

**DOI:** 10.3390/molecules22101755

**Published:** 2017-10-18

**Authors:** Lin Dong, Lei Yin, Hongfeng Quan, Yuankui Chu, Jincai Lu

**Affiliations:** 1 School of Traditional Chinese Materia Medica, Shenyang Pharmaceutical University, 103 Wenhua Road, Shenyang 110016, China; fanwuz716485@126.com; 2 School of Pharmacy, Ningxia Medical University, Yinchuan 750004, China; tangjuxian957@163.com (L.Y.); cisheng31352@yeah.net (H.Q.); 3 Department of Laboratory Medicine, General Hospital of Ningxia Medical University, Ningxia Medical University, Yinchuan 750004, China; lingshugc149179@126.com

**Keywords:** *Elaeagnus mollis* Diels, kaempferol-3-*O*-α-l-arabinopyranosyl-7-*O*-α-l-rhamnopyranoside, acute hepatic failure, apoptosis, inflammatory responses

## Abstract

Fulminant hepatic failure (FHF), associated with high mortality, is characterized by extensive death of hepatocytes and hepatic dysfunction. There is no effective treatment for FHF. Several studies have indicated that flavonoids can protect the liver from different factor-induced injury. Previously, we found that the extracts of *Elaeagnus mollis* leaves had favorable protective effects on acute liver injury. However, the role and mechanisms behind that was elusive. This study examined the hepatoprotective mechanisms of kaempferol-3-*O*-α-l-arabinopyranosyl-7-*O*-α-l-rhamnopyra-noside (KAR), a major flavonol glycoside of *E. mollis*, against d-galactosamine (GalN) and lipopolysaccharide (LPS)-induced hepatic failure. KAR reduces the mouse mortality, protects the normal liver structure, inhibits the serum aspartate aminotransferase (AST) and alamine aminotransferase (ALT) activity and decreases the production of malondialdehyde (MDA) and reactive oxygen species (ROS) and inflammatory cytokines, TNF-α, IL-6, and IL-1β. Furthermore, KAR inhibits the apoptosis of hepatocytes and reduces the expression of TLR4 and NF-κB signaling pathway-related proteins induced by GalN/LPS treatment. These findings suggest that the anti-oxidative, anti-inflammatory, and anti-apoptotic effects of KAR on GalN/LPS-induced acute liver injury were performed through down-regulating the activity of the TLR4 and NF-κB signaling pathways.

## 1. Introduction

Fulminant hepatic failure (FHF), also known as acute liver failure (ALF), is a serious liver injury caused by a variety of factors, such as viruses, drugs, poisons, alcohol, pregnancy, autoimmune liver diseases, hereditary metabolic disorder, cholestasis, etc., which is associated with high mortality and accompanied with hepatic encephalopathy, severe coagulation dysfunction, jaundice, or hydroperitoneum. In developing countries, viral infection is the most common cause of FHF, while in western countries, drug-induced acute liver injury has become the leading cause of FHF. The mechanism involved in pathogens or toxicants induced FHF includes two aspects. Firstly, pathogens or toxic substances directly damage the structure and organelles of liver cells and trigger series of cell signaling cascade, which lead to the disorders of cell structure and function, and then induce cell apoptosis or necrosis. Secondly, the damaged liver cells release endogenous damage-associated molecular patterns (DAMPs), which activates the self-immune system and stimulates immune cells to release inflammatory cytokines, which aggravates the damage of liver cells and finally results in fulminant hepatic failure [1]. Although great progress has been made in the pathogenesis and diagnosis of FHF, there is still no effective treatment. So far, the available treatment of FHF is liver transplantation, but it is limited by the shortage of donor livers [2]. Therefore, more and more researchers focus on looking for more effective medication of FHF [3,4,5,6,7].

*Elaeagnus mollis* Diels, as a traditional Chinese medicine, belongs to the Elaeagnaceae family and originates from the Tertiary Period, and is a deciduous dwarf tree or shrub that is an endangered, endemic species of China. This species is mainly distributed at the hills and the northern foot of the Qinling Mountains in the south of Shanxi province [8]. Previous studies found that the fruits of *E. mollis* could be used for anti-hypertension and anti-hyperlipemia, the seeds could be used for anti-oxidation and anti-consenescence, and the leaves could be made into a tea that has anti-inflammatory effects [9,10]. However, the main compounds and their pharmacological effect of *E. mollis* are still unclear.

In our previous study, we screened 70% ethanol extract of *E. mollis*, and its active constituents for hepatoprotective agents. Among them, kaempferol-3-*O*-α-l-arabinopyranosyl-7-*O*-α-l-rhamnopyranoside (KAR), a major active flavonol glycoside of *E. mollis*, showed hepatoprotective effects. In addition, several studies have demonstrated that flavonoids can protect the liver from different factors-induced injury by scavenging free radical, anti-oxidation, and anti-peroxidation of lipids and regulation of the immune system [11,12,13,14,15,16].

**Figure 1:**
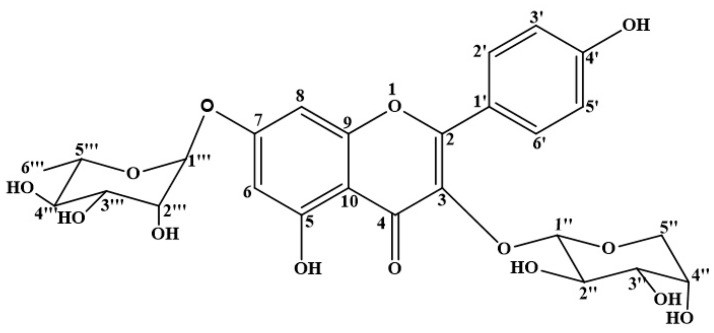
The chemical structure of KAR.

The purpose of this study was to elucidate the role and mechanism of KAR in LPS and GalN-induced acute liver injury. In this study, we extracted and purified KAR, with which we treated the animal model of liver injury established by the administration of GalN/LPS, and the liver damage, the statuses of oxidation, apoptosis, and inflammation were determined and compared in each treatment group as well as the control, as elucidated in Figure 1.

## 2. Materials and Methods

### 2.1. General

NMR experiments were performed on Bruker 400 FT-NMR spectrometers. TMS was used as an internal standard. High resolution electrospray ionization mass spectra (HR-ESI-MS) carried out on an Agilent Technologies 6224 TOF LC-MS apparatus. Mass spectra were recorded on a Bruker Daltonic micro TOF mass spectrometer. Silica gel (200–300 mesh, Qingdao Marine Chemical, Inc. Qingdao, Shandong, China) and Sephadex LH-20 (20–100 μm, GE Healthcare, Uppsala, Sweden); RP-ODS (50 μm) was purchased from Fuji Silysia Chemical Ltd. Silica. TLC was performed on pre-coated silica gel 60 F254 plates (20 × 20 cm^2^, 0.25 mm; Merck Chemicals Co., Ltd., Shanghai, China) and RP-18 F254 plates (20 × 20 cm^2^, 0.25 mm; Merck Chemicals Co., Ltd., Shanghai, China). The authentic sugar was bought from Sigma-Aldrich, Schweiz, Switzerland. Reagents were used of analytical grade and purchased from Yuwang Group Co., Ltd. (Yucheng, Shandong, China).

### 2.2. Plant Material

The leaves of *Elaeagnus mollis* Diels were collected from Xiangning county, Linfen city, Shanxi province, China, in October 2014 and identified by Professor Jincai Lu, who works at the School of Traditional Chinese Materia Medica, Shenyang Pharmaceutical University. Voucher specimens (EM20141001) were deposited in the herbarium of Shenyang Pharmaceutical University.

### 2.3. Extraction and Purification

The dry leaves of *E. mollis* (3.45 kg) were extracted using 35 L of 70% ethanol solution three times. Then, the extracts were filtered, concentrated under negative pressure, and suspended in distilled water (2.5 L). The resulting solution was partitioned successively to give petroleum ether (59.6 g), dichloromethane (36.0 g), *N*-butyl alcohol (103.5 g), and H_2_O (349.4 g) fractions. The n-BuOH fraction (103.5 g) was chromatographed in a 2000 g silica gel column, eluted with dichloromethane-methanol (40:1, 30:1, 20:1, 15:1,10:1, 8:1, 5:1, 1:1) to obtain nine fractions (F1–F9) based on TLC analyses. F6 (5.2 g) was separated by column chromatography (CC) over RP-ODS, eluted with methanol–water (2:8, 3:7, 4:6) to fractionize five fractions (F6-1 to F6-5). F6-3 (2.4 g) was applied to Sephadex LH-20 columns with methanol to yield compound **1** (yellowish needle-like crystals from methanol, 530 mg). Compound **1** was identified as KAR by comparing its spectroscopic data (^1^H-NMR, ^13^C-NMR) with values in the literature [17]. KAR was determined to be more than 98% pure by HPLC analysis.

### 2.4. Animals and Treatment

This study was approved by the animal care committee of Ningxia Medical University. Male ICR mice (18–22 g, license no. SYXK (NING) 2011-0001) were obtained from the experimental animal center of Ningxia Medical University, which were all SPF-grade animals and fed in a standard barrier environment. Twelve hourse after starvation, mice were intraperitoneally injected with 800 mg/kg d-galactosamine (GalN) (Sigma-Aldrich Chemical Co., St. Louis, MO, USA) and 40 μg/kg lipopolysaccharide (LPS) (Escherichiacoli O11:B4; Sigma-Aldrich Chemical Co., St. Louis, MO, USA). 150 mice were randomly divided into six groups. (a) control group: mice treated with sodium carboxymethyl cellulose (Na-CMC); (b) KAR control group: mice treated with 100 mg/kg KAR (purified dry KAR used in this study was suspended in water with 0.5% (*w/v*) Na-CMC); (c) FHF model group: mice treated with Na-CMC-dissolved GalN and LPS; (d–f) KAR treatment groups: mice treated with 25, 50, or 100 mg/kg bw, respectively. There were 25 mice in each group, 10 for survival observation, which were monitored for 24 h after GalN/LPS treatment, and the other 15 for biochemical and molecular detection.

Mice were orally administered Na-CMC or different concentration of KAR for seven days, and 60 min after the last administration, GalN and LPS were intraperitoneally injected. Six hours after GalN/LPS treatment, all the mice were sacrificed, the blood was collected from the retrobulbar vessels, and serum was separated by centrifugation (3000× *g*, 10 min), and kept at −80 °C. The liver samples obtained from the left lobe were washed with ice-cold phosphate-buffered saline (PBS) (Solarbio Science and Technology Co., Beijing, China) and divided into two parts; one was embedded in 4% paraformaldehyde (GuangNuo Chemistry&Science Technology Co., Shanghai, China) for histological assay and another was kept at −80 °C for the further study. The study protocol has been approved by the research ethical committee of our institute (no. 2014-103).

### 2.5. Histological Analysis and Detection of Apoptotic Cells (TUNEL Assay)

Liver samples fixed in 4% paraformaldehyde for two days were embedded in paraffin, serially sectioned, and stained with hematoxylin and eosin. Apoptotic cells were detected using TUNEL staining with an in situ apoptosis detection kit (Bioswamp, Wuhan, China) according to the manufacturer’s instructions. Finally, all liver sections were evaluated by pathologists under an Olympus BX51 microscope (Olympus, Tokyo, Japan) at 200× magnification.

### 2.6. Serum ALT/AST Analysis

Serum ALT/AST activity was determined by standard spectrophotometric procedures using the ChemiLab ALT/AST assay kit (Jiancheng Bioengineering Institute, Nanjing, China) according to the manufacturer’s instructions.

### 2.7. ELISA Assay

The levels of MDA, GSH, and ROS, and the production of TNF-α, IL-6, and IL-1β in mouse serum or liver lysates in each experimental group were measured using commercial ELISA kits (the kits for MDA, GSH, and ROS detection purchased from Shanhai Yuanye Biological Technology Co. Ltd., Shanhai, China, and the TNF-α, IL-6, and IL-1β detection kits were purchased from Neobioscience Biotechnology Company, Beijing, China) according to the manufacturer′s instructions.

### 2.8. Western Blot Assay

Total proteins of fresh liver tissues in each group were extracted using a whole cell lysis assay kit (KeyGEN BioTECH, Nanjing, China) and the protein concentration was determined using the bicinchoninic acid (BCA) protein quantitation assay kit (KeyGEN BioTECH, Nanjing, China). Equal amounts of lysates were separated by 12% SDS-PAGE gel electrophoresis and transferred to polyvinylidene fluoride (PVDF) membranes. After blocking in 5% (*w/v*) non-fat dry milk in PBST for 1 h, the membranes were incubated with primary antibodies for detecting cytochrome C, Bcl-2, Bax, caspase-3, caspase-8, caspase-9, TLR4, TRIF, TAF6, MyD88, p65, NF-κB, and Iκ-Bα (Cell Signaling Technology, Danvers, MA, USA) overnight at 4 °C. Then, the membranes were washed with TBST and incubated with HRP-conjugated secondary antibodies at room temperature for 1 h. Protein bands were visualized with ECL and determined using a chemiluminescence detection system equipped with imaging software (Bio-Rad Laboratories, Philadelphia, PA, USA).

### 2.9. Statistical Analysis

All data in this study was obtained from at least three independent experiments and presented as the mean ± standard deviation (SD). Statistical evaluation of the data was performed by one-way ANOVA when more than two groups were compared with a single control, and *t*-test for the comparison of differences between the two groups using SPSS 19.0 software. Significant differences were assigned to *p* values < 0.05 and < 0.01 denoted by * or ^#^ and ** or ^##^ respectively.

## 3. Results

### 3.1. Chemical Structure of KAR

Kaempferol-3-*O*-α-l-arabinopyranosyl-7-*O*-α-l-rhamnopyranoside. Yellowish powder, m.p. 210–212 °C, ESI-MS *m*/*z* 565.1428 [M + H]^+^ (C_26_H_28_O_14_H^+^, calcd. for 565.1429), ^1^H-NMR (400 MHz, DMSO) δ: 8.12 (2H, d, *J* = 8.8 Hz, H-2′,6′), 6.89 (2H, d, *J* = 8.9 Hz, H-3′, 5′), 6.83 (1H, d, *J* = 2.0 Hz, H-8), 6.45 (1H, d, *J* = 2.0 Hz, H-6),5.56 (1H, s, rha-H-1′′′), 5.36 (1H, d, *J* = 5.1 Hz, ara-H-1′′), 3.75 (1H, dd, *J* = 6.9, 5.5 Hz, H-2′′), 3.53 (1H, dd, *J* = 6.9, 3.0 Hz, H-3′′), 3.66 (1H, m, H-4′′), 3.58 (2H, dd, *J* = 11.5, 5.5 Hz, H-5′′), 3.21 (2H, dd, *J* = 11.4, 2.4 Hz, H-5′′), 3.85 (1H, br.s, H-2′′′), 3.64 (1H, dd, *J* = 9.4, 3.3 Hz, H-3′′′), 3.30 (1H, br.t, *J* = 9.4 Hz, H-4′′′), 3.43 (1H, dq, *J* = 9.3, 6.2 Hz, H-5′′′), 1.12 (3H, d, *J* = 6.1 Hz, rha-H-6′′′). ^13^C-NMR (101 MHz, DMSO) δ: 156.76 (C-2), 133.85 (C-3), 177.72 (C-4), 160.85 (C-5), 99.39 (C-6), 161.62 (C-7), 94.57 (C-8), 155.92 (C-9), 105.60 (C-10), 120.50 (C-1′), 131.11 (C-2′, 6′), 115.32 (C-3′, 5′), 160.26 (C-4′), 3-*O*-α-l-ara: 101.19 (C-1′′), 70.77 (C-2′′), 71.55 (C-3′′), 66.01 (C-4′′), 64.24 (C-5′′), 7-*O*-α-l-rha:98.37 (C-1′′′), 70.07 (C-2′′′), 70.27 (C-3′′′), 71.61 (C-4′′′), 69.81 (C-5′′′), 17.91 (C-6′′′). The above data were identical to the literature data [17]. Its chemical structure is shown in Figure 1.

### 3.2. KAR Reduces GalN/LPS-Induced Mortality

**Figure 2:**
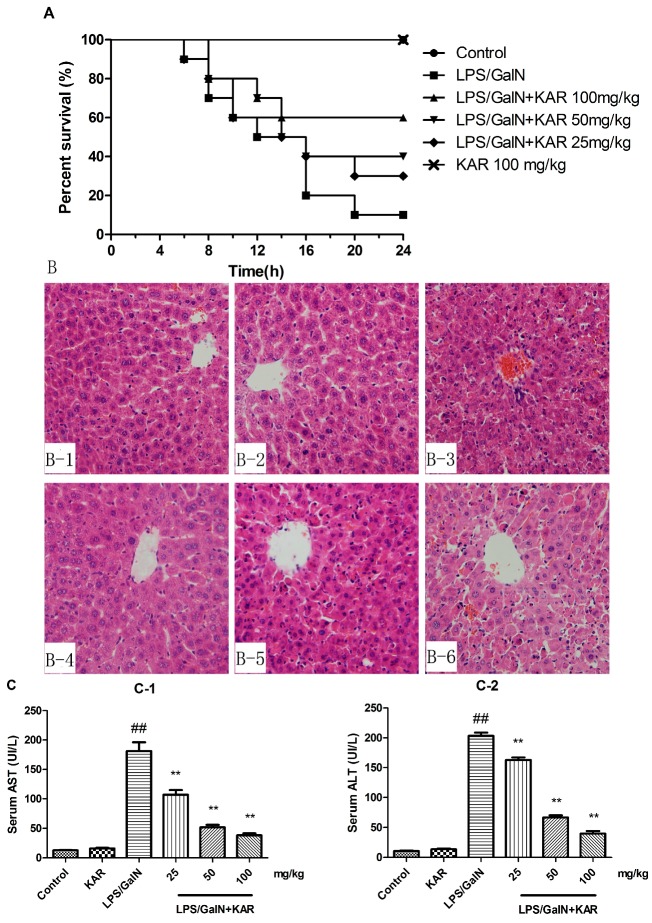
Effect of KAR on D-GalN/LPS-induced mouse acute liver injury. (**A**) Effect of KAR on the survival of mice in each group; (**B**) histopathological observation of liver tissue sections in control group (**B1**); KAR control group (**B2**); FHF model group; (**B3**) and KAR treatment group (**B4**–**6**) (magnification 200×); the activity of AST (**C1**) and ALT (**C2**) in serum was measured by assay. The data represented show the mean ± SD (*n* = 15). ** *p* < 0.01 compared to the FHF model group, and ## *p* < 0.01 compared to the control group.

To investigate the effect of KAR on GalN/LPS-induced mortality, the survival of mice within 24 h after GalN/LPS treatment was observed. We observed that mice began to die 6 h after GalN/LPS treatment, and their survival rate was 80% at 8 h and 10% at 20 h after GalN/LPS treatment in the FHF model group. Pretreatment with 25, 50, or 100 mg/kg KAR could increase the survival rate in a dose-dependent manner (Figure 2A). The survival rate of mice treated with 100 mg/kg KAR alone was unaffected.

### 3.3. KAR Protects the Normal Liver Structure and Decreases the Levels of Serum AST and ALT

To explore the effect of KAR on GalN/LPS-induced acute liver injury, the microstructure of livers was assessed by histological analysis. We found that the normal liver lobule structures were severely destroyed 6 h after GalN/LPS treatment, which included extensive hemorrhage, cellular apoptosis, and neutrophil infiltration. These pathological changes could be markedly alleviated by pretreatment with KAR (Figure 2B). Generally, significant elevation of serum AST and ALT levels are usually used as a marker of acute liver injury. Therefore, the levels of serum AST and ALT 6 h after GalN/LPS treatment was measured. Results showed that serum AST and ALT levels increased significantly after LPS/GalN treatment and this could be alleviated by pretreatment with KAR (Figure 2C). The liver microstructure and serum AST and ALT levels of KAR control group was unaffected.

### 3.4. KAR Decreases the Production of Peroxidation Products and Inflammatory Cytokines

**Figure 3:**
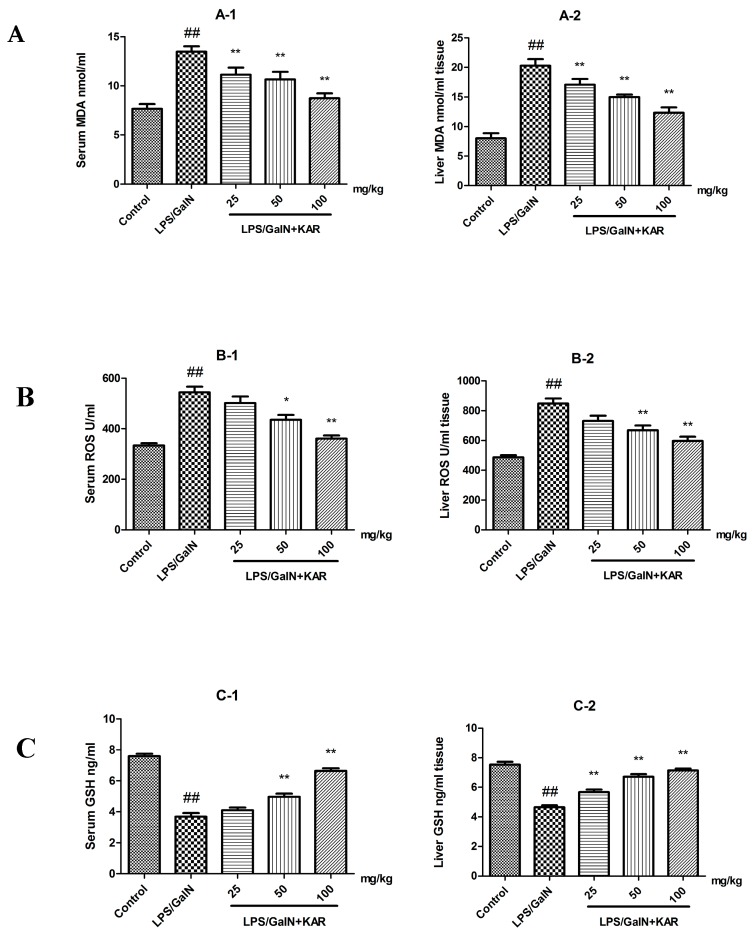
The levels of the MDA, ROS, and GSH (**A**–**C**) in serum and liver tissues were measured using an ELISA assay. The data represented show the mean ± SD (*n* = 15). * *p* < 0.05, ** *p* < 0.01 compared to the FHF model group, and ## *p* < 0.01 compared to the control group.

**Figure 4:**
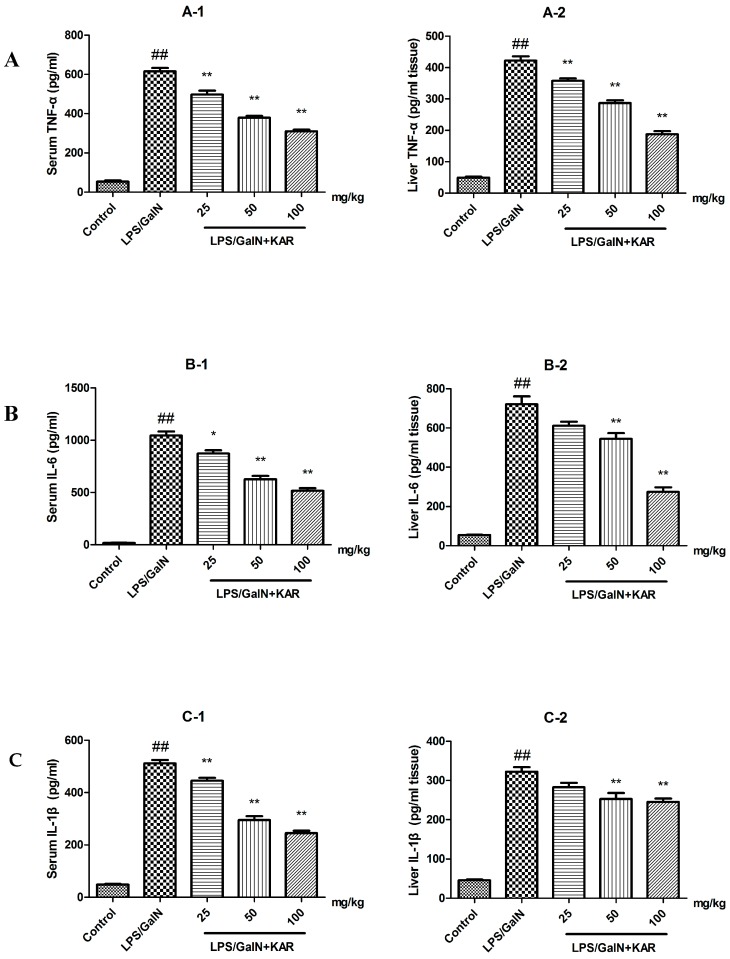
The production of TNF-α, IL-6, and IL-1β (**A**–**C**) in serum and liver tissues were measured using an ELISA assay. The data represented show the mean ± SD (*n* = 15). * *p* < 0.05, ** *p* < 0.01 compared to the FHF model group, and ## *p* < 0.01 compared to the control group.

Several studies have reported that flavonoids have antioxidant and anti-inflammatory effects in the treatment of various diseases. Meanwhile, we observed that KAR could alleviate GalN/LPS-induced neutrophil infiltration. In order to explore the mechanism behind that, the production of peroxidation products, MDA and ROS, and inflammatory cytokines, TNF-α, IL-1β, and IL-6, was evaluated by an ELISA assay. The results showed that the levels of MDA and ROS, and the production of TNF-α, IL-1β, and IL-6 in the serum and liver tissues, were significantly increased after GalN/LPS treatment, and this could be attenuated with pretreatment with KAR in a dose-dependent manner (Figure 3 and Figure 4). In addition, the level of GSH, which is an important antioxidant, was also detected. After treatment with GalN/LPS, the level of GSH significantly decreased in the serum and liver tissues and this could be reversed with pretreatment with KAR in a dose-dependent manner (Figure 3). These results suggest that KAR plays an important role in protecting against GalN/LPS-induced increases of peroxidation products and inflammatory cytokines.

### 3.5. KAR Inhibits GalN/LPS-Induced Apoptosis of Hepatocytes

**Figure 5:**
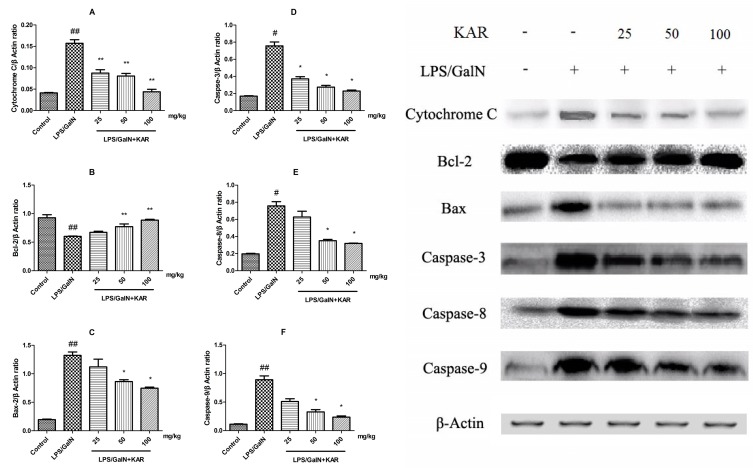
The expression of apoptotic factors, such as cytochrome C (**A**); Bcl-2 (**B**); Bax (**C**); caspase-3 (**D**); caspase-8 (**E**); and caspase-9 (**F**); was detected by immunoblotting of the liver tissues of each group 6 h after D-GalN/LPS administration. The data are normalized to that of β-actin and represent the mean ± SD; * *p* < 0.05, ** *p* < 0.01 compared to the FHF model group, and # *p* < 0.05, ## *p* < 0.01 compared to the control group.

**Figure 6:**
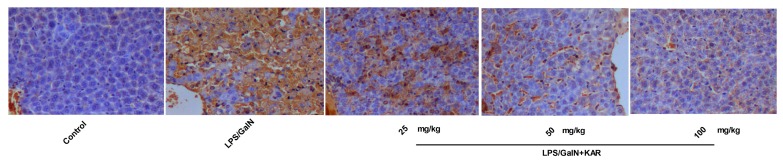
TUNEL assay was used to evaluate the effect of KAR on the in situ apoptosis in the liver control group (**1**), FHF model group (**2**), and KAR treatment group (**3**–**5**) (magnification 200×). LPS substantially induced apoptosis in liver, and the administration of KAR significantly improved it in a dose-dependent manner.

During histological analysis, we observed that KAR could alleviat GalN/LPS-induced apoptosis of hepatocytes. To further explore the role of KAR in hepatocyte apoptosis, the expression levels of apoptosis-related proteins, including cytochrome C, Bax, Bcl-2, caspases 3, caspase-8, and caspase-9 were detected using a Western blotting assay. Results showed the expression levels of mitochondrial apoptosis pathway-related proteins, including cytochrome C, Bax and caspase-9, meanwhile, the death receptor pathway-related protein, caspase-8, and the common pathway-related protein, caspase-3, increased significantly after GalN/LPS treatment compared with the control group, and this could be markedly reversed when pretreatment with KAR in a dose-dependent manner (Figure 5). In addition, the expression of Bcl-2, which is a promoting factor for cell survival, decreased significantly after treatment with GalN/LPS and increased markedly with pretreatment with KAR (Figure 5). Apoptosis has been observed on GalN/LPS-caused hepatic failure in animal models. In the study, apoptotic hepatocytes were detected with TUNEL staining. We observed that numerous TUNEL-positive hepatocytes in liver tissues obtained 6 h after GalN/LPS treatment. However, few positive hepatocytes were observed in livers treated with 50 mg/kg or 100 mg/kg KAR (Figure 6). These results suggest that the protective effects of KAR in GalN/LPS-induced acute liver injury might be regulated by both the death receptor pathway and the mitochondrial apoptosis pathway.

### 3.6. KAR Reduces the Expression of TLR4 and NF-κB Signaling Pathway Related Proteins

**Figure 7:**
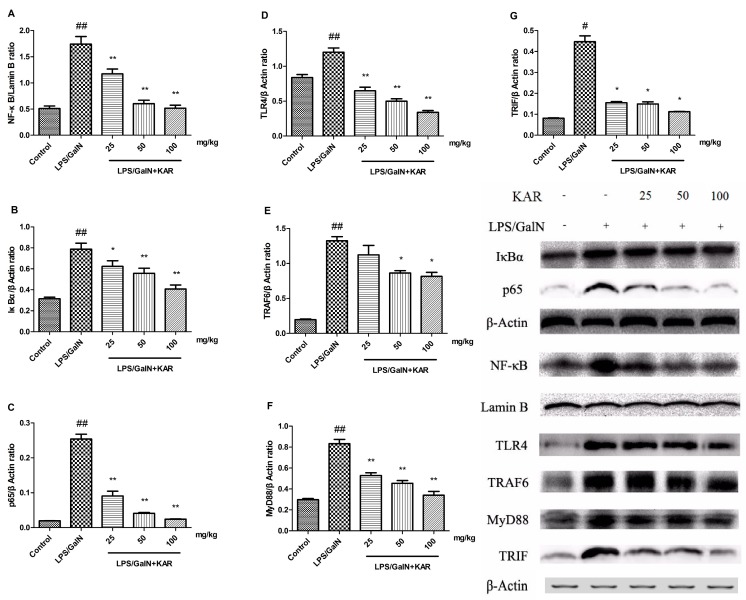
The expression of NF-κB and TLR4 signaling pathway-related proteins, such as NF-Κb (**A**); IκBα (**B**); p65 (**C**); TLR4 (**D**); TRAF6 (**E**); MyD88 (**F**); and TRIF (**G**); were detected using a Western blotting assay in the liver tissues of each group 6 h after D-GalN/LPS administration. The data represented show the mean ± SD. Data of IκBα, p65, and NF-κB are normalized to that of Lamin B, and that of TLR4, TRAF6, MyD88, and TRIF are normalized to β-actin. * *p* < 0.05, ** *p* < 0.01 compared to the FHF model group, and # *p* < 0.05, ## *p* < 0.01 compared to the control group.

To further investigate the protective mechanism of KAR in GalN/LPS-induced acute liver injury, the expression of TLR4 and NF-κB signaling pathway-related proteins in liver tissues was detected using a Western blotting assay. Results showed that the expression level of the NF-κB signaling pathway-related proteins, IκBα, p65, and NF-κB, and TLR4 signaling pathway-related proteins, TLR4, TRAF6, MyD88, and TRIF, increased significantly 6 h after GalN/LPS treatment compared with the control group, and this could be significantly reversed with pretreatment with KAR in a dose-dependent manner (Figure 7). These results suggest that the protective effects of KAR in GalN/LPS-induced acute liver injury is partially associated with the inhibition of TLR4 and NF-κB signaling pathway activity.

## 4. Discussion

The animal model of FHF could be established by intraperitoneal injection of d-galactosamine (GalN) and lipopolysaccharide (LPS) [18,19]. LPS, an endotoxin and a component of the bacterial cell wall, is usually used to induce the inflammatory response and the production of inflammatory cytokines. However, the sensitivity of mice to LPS is lower than that of humans. GalN is a hepatotoxic substance that inhibits the synthesis of RNA and protein in hepatocytes. LPS combined with GalN could enhance the sensitivity of animals to drugs [2,20] and activates the macrophages or hepatic Kupffer cells to produce pro-inflammatory cytokines, such as tumor necrosis factor (TNF)-α, IL-6, etc. Subsequently, these pro-inflammatory cytokines involved in the development of liver tissue injury [21,22]. Therefore, LPS combined with GalN was used to establish the mouse model of acute liver failure in this study. Significantly elevated serum levels of ALT and AST are often associated with severe pathological damage of liver and are usually used as markers of liver injury [20]. In this study, we found that 6 h after treatment with d-GalN and LPS, the serum levels of AST and ALT increased significantly, and the normal liver lobule structures were severely destroyed, which includes extensive hemorrhage, cellular apoptosis, and neutrophil infiltration. All these indicated that the mouse model of liver injury was established by intraperitoneal injection of 800 mg/kg GalN and 40 μg/kg LPS. To explore the effects of KAR on GalN/LPS-induced acute liver injury, the microstructure of livers, the survival of mice, and the levels of serum AST and ALT were assessed. Results showed that pretreatment with KAR could significantly reduce the mouse mortality, protect the normal liver structure, and decrease the levels of serum AST and ALT in a dose-dependent manner. These results suggested that KAR played important roles in GalN/LPS-induced acute liver injury.

Previous studies demonstrate that the protective effect of flavonoids against liver injury could be attributed to their ability to scavenge free radical, anti-oxidation, and anti-peroxidation of lipids and regulation of the immune system. Meanwhile, we observed that KAR could alleviated GalN/LPS-induced neutrophil infiltration. To further explore the hepatoprotective effects and its mechanism of KAR on GalN/LPS-induced acute liver injury, the production of peroxidation products and inflammatory cytokines was measured using an ELISA assay. Several studies reported that LPS could trigger numerous pathological events, including the production of inflammatory cytokines, such as TNF-α, IL-1β, and IL-6, and inhibition of the production of TNF-α, IL-1β, and IL-6 could alleviate the damage to the liver [23]. IL-6 is an important inducer of infection defense, crucial for hepatocyte homeostasis, and a potent mitogen of hepatocytes. The synthesis and secretion of IL-6 is induced by inflammatory responses [24]. IL-1β, a potent pro-inflammatory cytokine, produced by hepatocytes and neutrophils, can activate and recruit leukocytes, especially neutrophils, into the liver. Then, the activated neutrophils act as effector cells, which can induce the necrosis of hepatocytes through cytotoxicity [25]. TNF-α, which can induce the necrosis of hepatocytes and the production of other cytokines, such as IL-1β and IL-6, plays a vital role in the pathogenesis of liver injury. Oxidative stress and lipid peroxidation, which can trigger the production of inflammatory cytokines and, subsequently, cell death and tissue damage, are involved in the occurrence of liver injury [26,27]. MDA, the final degradation product of lipids caused by oxidative stress, is used as a marker of oxidative stress and can reflect the degree of cell damage induced by oxidative stress [25,28]. ROS, involved in the pathogenesis of liver cirrhosis, is also a risk factor for liver cancer [29]. The level of GSH, used as a sign of hepatotoxicity, is higher in healthy mice than that of mice with liver diseases [30]. Therefore, the expression of inflammatory cytokines, TNF-α, IL-1β, and IL-6, and the levels of MDA, ROS, and GSH, markers of oxidative stress, in serum and liver tissues was detected in this study. Results showed that the levels of MDA and ROS and the production of TNF-α, IL-1β, and IL-6 in the serum and liver tissues were significantly increased after GalN/LPS treatment, and this could be attenuated by pretreatment with KAR. In addition, we found that pretreatment with KAR can increase the level of GSH, which is an important antioxidant. These results suggest that KAR plays an important role in protecting against GalN/LPS-induced inflammatory responses and oxidative stress.

Previously, we observed that GalN/LPS treatment could induce the apoptosis of hepatocytes, and it is demonstrated that dysregulation of apoptosis is associated with various pathological conditions, such as cancer, autoimmune diseases, neurodegenerative diseases, and acute hepatic failure [31,32,33,34,35,36]. Thus, we suggested the apoptosis of hepatocytes that induced by GalN/LPS treatment is involved in the development of liver injury. There are two major apoptotic pathways: the extrinsic pathway triggered by the activation of caspase-8 and the intrinsic pathway triggered by the activation of caspase-9. Activated caspase-8 and caspase-9 can directly activate downstream caspases, such as caspase-3, and induce the releasing of cytochrome C from mitochondria, which can finally lead to the apoptosis of cells [37,38]. The Bcl-2 family, including pro-apoptotic proteins, such as Bax, and anti-apoptotic proteins, such as Bcl-2, play important roles in the regulation of apoptosis [38]. To further explore the role of KAR in hepatocyte apoptosis, the expression level of apoptosis-related proteins, including cytochrome C, Bax, Bcl-2, caspase-3, caspase-8, and caspase-9 were detected. Results showed the expression level of the intrinsic apoptotic pathway-related proteins, including cytochrome C, and caspase-9, meanwhile, the extrinsic apoptotic pathway related proteins caspase-8 and the common pathway-related proteins caspase-3, increased significantly after GalN/LPS treatment, and this could be markedly reversed with pretreatment with KAR in a dose-dependent manner. In addition, the expression of Bcl-2 decreased significantly and the Bax expression increased significantly after GalN/LPS treatment, and pretreatment with KAR could reverse this. These results suggest that KAR can inhibit GalN/LPS-induced intrinsic and extrinsic apoptosis by regulating the expression of Bcl-2 and Bax.

To further identify the mechanism underlying the anti-inflammatory and anti-apoptotic effects of KAR in LPS-induced liver injury, the NF-κB and Toll-like receptor 4 (TLR4) pathway-related proteins were detected. NF-κB, which can be activated by several inflammatory cytokines, such as TNF-α, IL-1β, and IL-6, regulate the expression of numerous genes that are involved in immune responses, inflammatory responses, apoptosis, and tumorigenesis [39]. NF-κB is the dimer of p50 and p65. When inactivated, NF-κB, together with IκB, forms an inactive complex in the cytoplasm. When IκB is phosphorylated and dissociated from the inactive complex, NF-κB is activated and translocates into the nucleus, alternating the transcription efficiency of multiple target genes [40]. In this study, we found that after GalN/LPS treatment, the expression of inflammatory cytokines and NF-κB pathway-related proteins, NF-κB, p65, and IκBα increased significantly and this could be reversed by pretreatment with KAR. It is demonstrated that inhibition of the expression of NF-κB can decrease the production of pro-inflammatory cytokines [41]. Therefore, our results suggested that the anti-inflammatory effects of KAR were associated with the inhibition of NF-κB. Toll-like receptors (TLRs), a family of proteins that control the innate immune responses [42], are widely expressed on parenchymal and non-parenchymal cells in the liver [43]. TLR4, a recognition receptor of LPS, is involved in the pathogenesis and progression of several liver diseases, including alcoholic and non-alcoholic steatohepatitis, fibrogenesis, and inflammatory diseases of the liver [44,45,46]. Recently, it was reported that TLR4 plays important roles in GalN/LPS-induced liver injury, and inhibiting the activity of TLR4 signaling is a potential therapy [47,48]. LPS-mediated TLR signaling consists of two parts, the MyD88-dependent pathway and the MyD88-independent pathway [49]. To determine the role of TLR4 signaling in the hepatoprotective effect of KAR, the expression of the TLR4 pathway-related proteins, TLR4, MyD88, TRIF, and TRAF6, was detected. MyD88, an essential adaptor protein that integrates and transduces intracellular signaling generated by TLRs [46], can activate the transcription of NF-κB [42] and increase the expression of pro-inflammatory cytokines [50]. TRIF, another adaptor protein of the TLR pathway, is mainly responsible for the regulation of the MyD88-independent pathway [51]. TRAF6 is associated with TAK-1, which mediates the activation of NF-κB [49]. Our results indicated that KAR inhibited GalN/LPS-induced activation of TLR4 signaling. It is suggested that KAR can inhibit TLR4-mediated inflammatory responses.

## 5. Conclusions

In summary, we isolate kaempferol-3-*O*-α-l-arabinopyranosyl-7-*O*-α-l-rhamnopyranoside (KAR), a major flavonol glycoside from the leaves of *E. mollis*, which shows anti-oxidative, anti-inflammatory and anti-apoptotic effects on GalN/LPS-induced acute liver injury through down-regulating the activity of TLR4 and the NF-κB signaling pathway. Therefore, KAR can be used as a potential medication for preventing hepatic failure, and further studies should focus on the pharmacological and pharmacodynamic effects of KAR.
